# Pulsating Exophthalmos

**Published:** 1890-05-03

**Authors:** 


### PULSATING EXOPHTHALMOS.

Dr. Eissen, of Bern, reports a somewhat unusually severe case of this lesion, cured by repeated ligation of the common carotid and several of its branches. A young woman, aged 21, who was said to have had "dropsy" when in her fifteenth year, but who had enjoyed good health since, barring some irregularity of her periods, having a few days before one of these taken a long and fatiguing walk in the hot weather of July, was seized with vomiting which recurred during the evening. In one of these attacks she suddenly lost the sight of the right eye, which was extruded, with such a noise in both ears as to render her nearly deaf to every other sound. Two leeches were at once applied, but with no benefit, though the bleeding was arrested with difficulty. When seen by Dr. Eissen, the right eyeball was greatly displaced, directly forwards, perfectly immovable, extraordinarily hard to the touch, and apparently flattened out by pressure from behind. The upper lid was œdematous and carried forwards with the globe; the lower everted, chemotic, and of a purplish-red colour. The ophthalmoscope revealed atrophy of the optic papilla, its pale attenuated vessels scarcely extending beyond its margin. The globe itself did not pulsate, but pulsation was evident in the inner angle of the upper lid. At this point a loud murmur was distinctly audible, and blowing sounds over the temples and occiput. Pressure on the right carotid caused the pulsation and murmurs to cease, though the blowing sounds and the subjective noises in the ears remained unaffected by the compression.

Next day an erosion appeared on the everted lower lid, from which a fine jet of distinctly pulsating and arterial blood issued with considerable force. The common carotid artery was at once ligatured; the pulsation and murmurs ceased immediately, but the exophthalmos persisted as before. About two weeks later the tension of the ball was perceptibly less, and a certain amount of movement was possible, the pupil contracted when a strong light was cast on the sound eye, the everted lower lid resumed its normal position, and the œdema of the upper lid was less. At the end of a month the ball could be pushed back, but in so doing a pulsation was felt, while murmurs were again audible over the ball and the margins of the orbit.

The patient thus relieved was discharged, but she returned five weeks later, complaining greatly of buzzing in her right ear, which could be heard objectively along with the previously audible blowing sounds on that side of the head. Next month the murmurs returned with exaggerated intensity, the exophthalmos had increased to the extent of 15 mm., and the veins of the globe were rapidly swelling. The right internal carotid was now tied, and finding that the portion of the common carotid above the point of the previous ligature still pulsated, Dr. Eissen tied also successively the superior thyroid, the external carotid, the ascending pharyngeal, and the common carotid above the previous ligature, thus effectually limiting the collateral circulation on all sides. All sounds, objective and subjective, now ceased, the blind eye was removed later, and the patient has since remained free from any discomfort.

